# Effectiveness of health education interventions to improve malaria knowledge and insecticide-treated nets usage among populations of sub-Saharan Africa: systematic review and meta-analysis

**DOI:** 10.3389/fpubh.2023.1217052

**Published:** 2023-08-03

**Authors:** Opara Monica Onyinyechi, Ahmad Iqmer Nashriq Mohd Nazan, Suriani Ismail

**Affiliations:** Department of Community Health, Faculty of Medicine and Health Sciences, Universiti Putra Malaysia, Serdang, Malaysia

**Keywords:** malaria, health education, insecticide-treated nets, sub-Saharan Africa, meta-analysis

## Abstract

**Introduction:**

Malaria health education intervention is a community-directed approach that has long been considered important in preventing malaria in sub-Saharan Africa. However, its effectiveness is being questioned due to a lack of strong evidence. We aim to synthesize the evidence of the impact of health education on malaria knowledge and insecticide-treated nets (ITN) usage. Specifically, we analyzed the odds of correctly answering malaria-related questions and the odds of using ITN between the intervention and control groups.

**Methods:**

Experimental and observational studies conducted in sub-Saharan Africa between 2000 and 2021 which had quantitatively evaluated the impact of health education interventions on malaria knowledge and ITN usage were included in the review.

**Results:**

A total of 11 studies (20,523 participants) were included. Four studies used educational interventions to teach appropriate ITN strategies and promote ITN usage. Two others focused on improving knowledge of malaria transmission, prevention, treatment, and its signs and symptoms. The remaining five studies assessed both ITN use and malaria knowledge. Of these, 10 were eligible for meta-analysis. On average, the odds of a person in the intervention group reporting better malaria knowledge (odds ratio 1.30, 95% CI: 1.00 to 1.70, *p* = 0.05) and higher ITN usage (odds ratio 1.53, 95% CI: 1.02 to 2.29, *p* = 0.004) increased significantly after receiving health education interventions compared to those in the control group. The odds of ITN usage also substantially increased when the interventions were based on a theory or model (odds ratio 5.27, 95% CI: 3.24 to 8.58, *p* = 0.05).

**Discussion:**

Our review highlights sub-Saharan Africa’s various health education strategies to curb malaria over the past two decades. Meta-analysis findings show that health education interventions are moderately effective in improving malaria knowledge and ITN usage and have contributed to the effort of global malaria strategy.

## Introduction

Malaria poses a public health challenge in endemic African countries. The World Malaria Report estimated that there were 228 million cases of malaria in 2020 in the African Region, an increment of 15 million cases from 2019 (1). The number of deaths also showed an upward trend between 2019 and 2020, from 534,000 to 602,000 (1). About 95% of the disease burden continues to be borne by the African countries and the region also account for most of the increases in cases (1).

Widespread poverty and inadequate economic policies are among the factors contributing to the rising threat of malaria in these countries (2). Poor living circumstances coupled with ideal climatic conditions create the ideal setting for the reproduction and propagation of female anopheles mosquitoes, and people living in such areas often have little or no access to adequate malaria treatment (3, 4). Arguably, transmission levels may not be very different from 50 years ago in certain parts of central and west Africa (5).

Concerted efforts have been made in drug development, diagnosis, and control measures to eliminate and eradicate the disease. Determining the endemic level of human malaria infection in populations exposed to the disease is crucial to formulating specific and appropriate intervention strategies. Malaria transmission can occur all year round or in certain months of the year. Knowledge of malaria transmission patterns in every epidemiological setting is essential for appropriately selecting and planning intervention strategies and monitoring.

Insecticide-treated nets (ITN) are well-known malaria prevention devices recommended by the World Health Organization (WHO) for people living in malaria-endemic countries, especially those at risk of acquiring the disease (6). WHO defines ITN as “mosquito nets that repel, disable or kill mosquitoes that come into contact with the insecticide on the netting material (7).” ITN have been attributed to the decline of malaria prevalence in endemic sub-Saharan Africa by 50% between 2001 and 2015 (7). Malaria mortality rates have also decreased by almost 60% with ITN usage from 4.7 to 1.9 deaths per 10,000 people annually (8). Such worldwide progress in the fight against malaria can partly be attributed to the implementation of evidence-based interventions where malaria vector control has been the central component (8, 9).

Bhatt et al. (10) found that about 81% of malaria cases prevented in African countries since early 2000 were due to effective vector control interventions through the large-scale distribution and use of ITN and indoor residual spraying (IRS) of insecticides inside residential homes. In countries where malaria control efforts are confronted with political instability and complex and intense transmission cycles, ITN and IRS, along with improved malaria case management, have contributed to the decreasing trends in all-cause child mortality observed over the last 10 years (5, 11).

Global efforts in malaria control have primarily geared towards the utilization of ITN. However, utilization of ITN has plateaued since 2016 in the African region involving 40 countries (12). Additionally, in a region with high malaria transmission, ITN usage has proven to be the most cost-effective malaria prevention intervention (13, 14), and therefore increasing ITN use is the most promoted malaria vector control strategy in sub-Saharan malaria endemic countries (6, 14, 15).

Lack of access to ITN, excessive heat and ventilation discomfort side-effects, poor ITN perception (i.e., dislike by spouses, high cost), and lack of knowledge on malaria and ITN have been previously reported as significant barriers to its adoption in parts of Africa (16–18). Additionally, wealthy people living in urban areas with higher education levels are more likely to own and use ITN than those living in rural areas with low levels of education (19), exacerbating the disproportionate malaria impact on the poor.

There are also ITN improper usage and care issues stemming from behavioral factors. It was reported that some households misused ITN as alternatives for curtains and bed sheets, while a few individuals sold it off for a quick financial fix (20). Others blamed the traditional house’s windowless and space-limited characteristics that hindered them from properly installing ITN to avoid accumulating too much soot and dirt within the house (20).

Malaria health education interventions have shown some potential in improving malaria-related knowledge and ITN usage through multiple tailored curriculums and delivery methods (21–31). However, the pooled estimates of the effect of these interventions on participants’ knowledge and ITN utilization rate have yet to be discovered. Therefore, a quantifiable summary of health promotion and education interventions focusing on improving malaria knowledge and the frequency of ITN use is warranted. Our review aims to evaluate the effectiveness of current health education strategies among the population of sub-Saharan African countries.

## Materials and methods

This systematic review was performed per a pre-specified protocol and is reported according to the Preferred Reporting Items for Systematic Reviews and Meta-analysis (PRISMA) statement (32).

### Criteria for study selection

- **Types of studies.** Our systematic review included studies that reported the application of health education interventions in increasing ITN use and malaria knowledge.
- **Population.** The studies were carried out among people and communities of sub-Saharan African countries.
- **Research design.** Study designs considered were randomized controlled trials (RCT), cluster RCT (c-RCTs), quasi-experimental design, pretest-posttest, and cross-sectional survey. The review was guided by the Evidence for Policy and Practice Information and Coordinating Centre Methods for Conducting a Systematic Review (EPPI) (33). The EPPI method was chosen over the conventional systematic reviews (e.g., Cochrane method) because it allowed for a better variety of narrative synthesis and study designs to be included (34) rather than just randomized controlled trials (RCTs) if the latter was used.
- **Outcome measures.** The studies measured either improvement in malaria knowledge, ITN utilization, or both. Studies on malaria prevention and control interventions without a health education component were excluded.
- **Language.** The literature search was restricted to publication in English.
- **Timeframe.** The start of the time frame considered for the review was the year the milestone in primary healthcare to achieve health for all was set, which is the year 2000, as declared during the Alma Ata Conference organized by United Nations in 1978 (35). The Alma-Ata conference emphasized that educating the public on the prevention and control of health problems and encouraging participation, prevention, and management of locally endemic diseases as several of its main plan of action for primary healthcare (36). Updates on new publications were obtained weekly until no new study was identified that fit our inclusion criteria at the time this manuscript was written.

### Search strategy

We used several complementary approaches, including searches of the traditional and grey works of literature, forward and backward reference harvesting, and hand searching of targeted journals. A systematic search was conducted using seven (7) electronic databases; Science Direct, CINAHL, PubMed, Prisma, Pico, Cochrane Library, and PsycINFO. These seven databases are known to hold an extensive collection of journals in allied health sciences and public health. Other websites such as Malaria Journal, World Health Organisation (WHO), and Centre for Disease Control and Prevention were also searched. This was done to get studies not published in the primary databases to reduce selection bias. Key search terms used in each electronic search engine were as follows: “health education,” “ITN use*,” “malaria,” “general populations,” and “sub-Saharan Africa.” Boolean operators such as “OR/AND” were used to broaden or narrow the search. For instance, ITN OR other ITN synonyms (i.e., long-lasting treated net, mosquito net OR bed net), “malaria AND health education”, “malaria AND sub-Saharan Africa”, “health education OR health promotion.” Word truncation was also employed; thus, words like “malaria” and “health education” were written as “malaria*” and “health education*.” The full list of key terms used for the literature search is included in the Supplementary material.

### Data collection process

- **Abstract screening.** We used a thorough methodology to screen the large number of studies identified during this round. We developed an abstract screening guide (see Supplementary material), and all team members were involved in reviewing the abstracts. Reliability was ensured through a series of discussions to cross-check extracted abstracts following independent analysis by the authors.
- **Full-text retrieval.** Full-text manuscripts were retrieved for all the abstracts deemed eligible by a minimum of two authors in the previous round. They were then screened again against the inclusion and exclusion criteria.
- **Data extraction and synthesis.** Data were extracted using a standardized method created by the Centre for Reviews and Dissemination (37). Checklists for data extraction were drafted, pilot-tested, and refined at the protocol stage to minimize bias and improve reliability and validity. The checklists consisted of publication details, the aim of the study, study design, intervention components, study population, implementation method, time/year of intervention exposure, and study outcomes.
- **Study quality assessment.** The assessment of the quality of selected studies was guided by a tool designed by the Effective Public Health Practice Project (EPHPP) (38). Eight categories were assessed, which include study design, selection bias, confounders, blinding, data collection methods, attrition, intervention integrity, and analyses (see Table 1).

**Table 1:** Overview of the studies.

Study (year)/country	Study objectives	Study design	Intervention components	Study population	Implementation methods	Time of intervention exposure	Study outcomes	Study quality
Rhee et al. (2005)/Mali	To compare ITNs use and malaria knowledge levels between households who received the educational intervention and those who did not	RCT	Health education curriculum	Community members	Skill training and education about signs, symptoms, susceptibility, transmission, and prevention of malaria as well as proper ITN use	Not stated	ITN use greater in intervention group (48%) vs. control (33%, OR = 1.9, *p* = 0.012). No difference was found in knowledge between the intervention (change score = 2.14) and control groups (change score = 2.12).	SB: Strong SD: Strong C: Moderate B: Moderate DCM: Strong W&DO: Moderate GR: High quality
Cox et al. (2018)/South Africa	To evaluate the community-led Malaria Awareness Program (MAP) in increasing malarial knowledge	RCT	Health education curriculum with capacity building approach	Community members	Education workshops led by home-based care workers	Three- or four-week intervention	The adjusted odds in knowledge score between individuals who completed MAP (*n* = 499) vs. individuals who did not complete MAP (*n* = 399) was 3.3 and 2.8 times greater for transmission and prevention, respectively (*p* < 0.001)	SB: Strong SD: Strong C: Moderate B: Moderate DCM: Strong W&DO: Moderate GR: High quality
Ayi et al. (2010)/Ghana	To determine the impact of school-based malaria education intervention in school children and community adults	RCT	Health education curriculum	Community members	Skill training and education about signs, symptoms, susceptibility, transmission, and prevention of malaria as well as proper ITN use *via* teachers.	12 months	The misperception that malaria has multiple causes significantly improved among children and community adults in the intervention group. The community adults who treated a bed net with insecticide in the past 6 months increased from 21.5% to 50.0% (*p* < 0.001) in the intervention group	SB: Strong SD: Strong C: Moderate B: Moderate DCM: Strong W&DO: Moderate GR: High quality
De La Cruz et al. (2009)/Ghana	To compare the knowledge and behaviors of microfinance clients receiving malaria education (*n* = n = 213) to those receiving diarrhea education (*n* = n = 223) and non-client controls (*n* = n = 268)	c-RCT	Health education curriculum	Women of reproductive age (15–-49 years) with a child under five	Skill training and education about signs, symptoms, susceptibility, transmission, malaria prevention, and proper ITN use	Not stated	Malaria clients had significantly better malaria knowledge than comparison groups (diarrhea clients and non-clients). Malaria clients also experienced the most significant increases in ITN ownership/use (9% vs. 2.9% and 6.7% among diarrhea clients and non-clients)	SB: Strong SD: Strong C: Moderate B: Moderate DCM: Strong W&DO: Moderate GR: High quality
Deribew et al. (2012)/Ethiopia	To determine the effectiveness of skill-based training for household heads on ITN utilization	c-RCT	Training of household heads on the proper use of ITNs	Community members	Skill training and education about signs, symptoms, susceptibility, transmission, malaria prevention, and proper ITN use	Not stated	81.0% of individuals in the intervention villages and 79.3% in the control villages had utilized ITN at the six-month follow-up compared to 47.9% (intervention) and 68.4% (control) at baseline	SB: Strong SD: Strong C: Moderate B: Moderate DCM: Strong W&DO: Moderate GR: High quality
Amoran et al. (2012)/Nigeria	To assess the effect of health education on the uptake of ITN among nursing mothers in rural communities in Nigeria	Quasi design	Health education curriculum	Nursing mothers	Training workshops, the use of educational materials such asposters, story books, and malaria post signs	2 weeks	The ITN users in the experimental group were 59 (29.5%) and 138 (72.6%) in the pre- and post-intervention period, respectively (*p* = 0.0001), while ITN users in the control group were 55 (27.5%) and 57 (31.6%) in pre-post intervention periods (*p* = 0.37)	SB: Moderate SD: Strong C: Moderate B: Moderate DCM: Strong W&DO: Weak GR: Moderate quality
Keating et al. (2012)/Zambia	To evaluate the effect of a community health worker (CHW)—based, interpersonal communication campaign (IPC) for increasing insecticide-treated mosquito net (ITN) use	Quasi design	Health education curriculum	Children under 5 years old	House-to-house visits, visual aids, pictures, printed leaflets, community plays, and demonstrations	2008–2010, four times per year	There was no indication that the CHW-based intervention activities had a significant effect on increasing ITN use (*p* > 0.05)	SB: Moderate SD: Strong C: Moderate B: Moderate DCM: Strong W&DO: Weak GR: Moderate quality
Afolaranmi et al. (2015)/Nigeria	To assess the knowledge of malaria and long-lasting insecticide treated nets (LLITNs) among people living with HIV/AIDS	Quasi design	Health education curriculum	Community members	Skill training and education about signs, symptoms, susceptibility, transmission, and malaria prevention	Not stated	The malaria knowledge improved significantly after the training (*pP* < 0.001). The majority (98.8%) of the respondents had good knowledge of LLITNs after the intervention (*p* < 0.001)	SB: Moderate SD: Strong C: Moderate B: Moderate DCM: Strong W&DO: Weak GR: Moderate quality
Abamecha et al. (2021)/Ethiopia	To examine the effectiveness of the school-based SBCC approach on insecticide-treated nets (ITNs) utilization among primary school students in malaria endemic	Quasi design	School-based social and behavior change communication (SBCC) approach	Primary school students and various community groups, including vulnerable groups such as children under 5 years and pregnant women	Peer education, capacity building, participatory consultations, educational sessions	2017 to 2019	ITNs utilization was 6.857 folds in the intervention groups compared to the counterpart: [OR = 6.857; 95% CI: (4.636, 10.1430); effect size = 39%]	SB: Moderate SD: Strong C: Moderate B: Moderate DCM: Strong W&DO: Weak GR: Moderatequality
Kebede et al. (2020)/Ethiopia	To develop, implement and evaluate the effects of school-based malaria social behavior change communication (SBCC) in terms of community message exposure, acceptance, knowledge, and practices	Pretest posttest	School-based social and behavior change communication (SBCC) approach	Primary school students and community groups	Peer education, capacity building, participatory consultations, educational sessions	17 months	Comprehensive knowledge about malaria increased to 39.1% (effect size = 14.8%). Insecticide-treated nets (ITNs) usage was improved to 63.0% (effect size = 25.8%)	SB: Moderate SD: Moderate C: Moderate B: Moderate DCM: Strong W&DO: Weak GR: Moderate quality
Nwachukwu et al. (2019)/Nigeria	To investigate the influence of media messages on ITN and artemisinin combination therapy use	Cross-sectional survey	Mass media messages on ITN and ACT use	General population	A structured questionnaire	Not stated	Exposure to malaria messages did not result in population-wide adoption of ITN and ACT	SB: Moderate D: Weak C: Weak B: Moderate DCM: Moderate W&DO: Weak GR: Low quality

### Meta-analysis

Two separate meta-analyses were performed to determine the effectiveness of interventions in improving malaria knowledge and ITN use. We used ReviewManager (RevMan 5.4) built-in variance correction to calculate 95% confidence intervals to reflect the heterogeneity estimates. In this review, any statistical heterogeneity would be acceptable, and any estimates of the average effect of interventions were worth reporting. Statistical heterogeneity of the included studies was explored using the *I*^2^ statistics. Odd ratios were analyzed using the random effects option within the RevMan 5.4 program.

## Results

### Search outcomes

The search yielded 683 studies, with five additional records identified through reference-list checking (*n* = 3) and personal communication with experts (*n* = 2). 571 abstracts were excluded during the screening process for failing to meet one or more inclusion criteria. The remaining 117 studies were reviewed as full text. Of these, 103 more studies were excluded following a detailed assessment of each of the intervention approaches (i.e., did not have malaria knowledge/ITN usage as the outcome; did not include health education component) and three others after obtaining further information from the study authors (i.e., two were still ongoing and one required supplementary data that did not receive clearance for release). Eleven studies met eligibility criteria and were included in the final review, 10 of which were evaluated further through meta-analysis.

### Study designs

There were three RCTs (21–23), two c-RCTs (24, 25), four quasi-experiments (26–29), one pretest-postest (30) and one cross-sectional (31) out of the 11 studies reviewed. As shown in Table 1, Ethiopia and Nigeria each had three studies conducted within their respective territories, while Ghana had two. Mali, South Africa, and Zambia each recorded one study, respectively.

### Study participants

Study participants covered in this review were enrolled from sub-Saharan African countries. They can be categorized into adults enrolled during household visits, heads of households (men), pregnant women with children under 5 years old, and nursing mothers.

### Study characteristics

**Figure 1:**
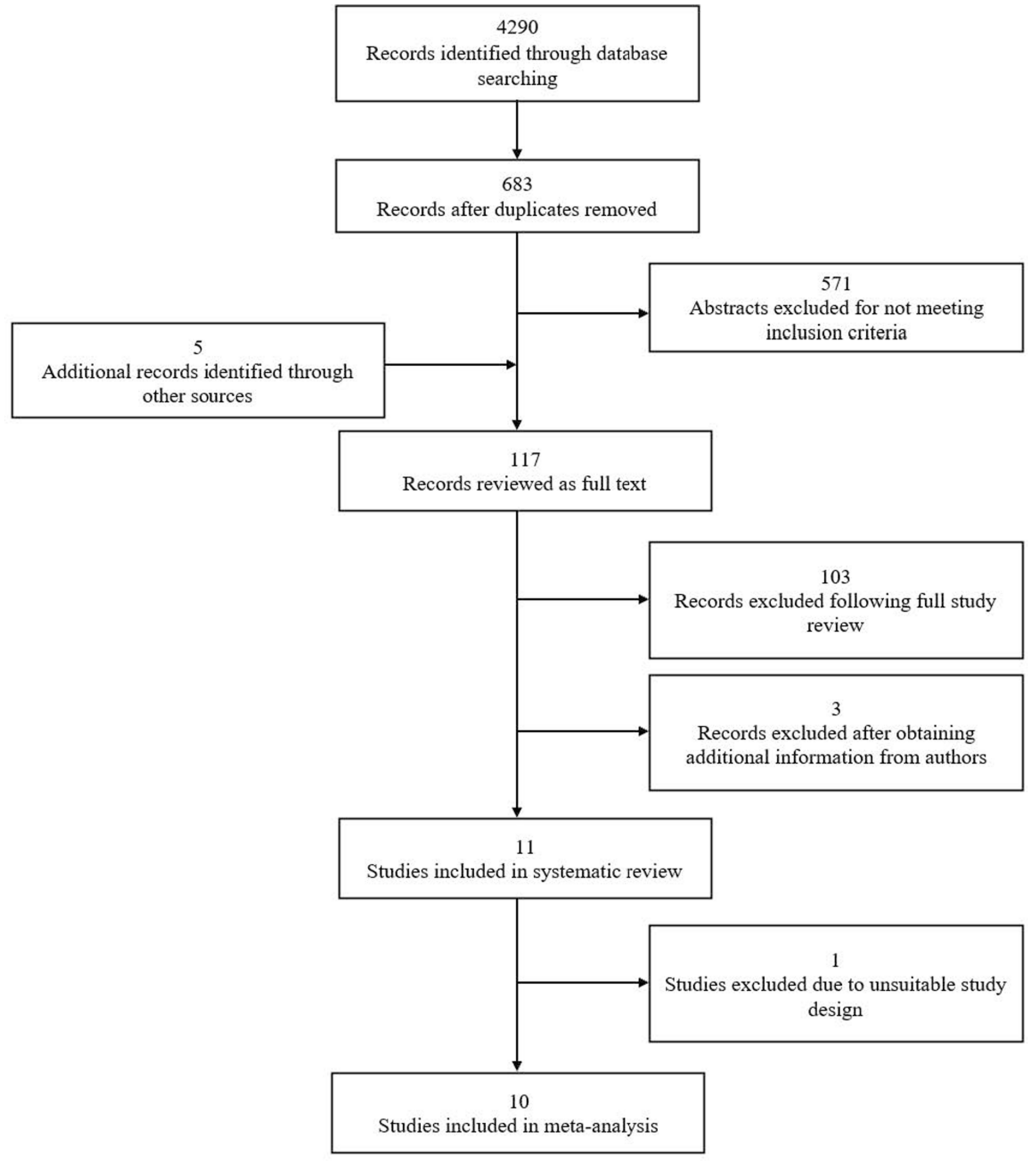
PRISMA flow diagram of records/studies included at each stage of screening.

In reviewing the studies (see Figure 1), four used educational interventions to teach appropriate ITN strategies and promote its usage (21–23, 25), two other studies examined knowledge and practices regarding malaria transmission, prevention and treatment, and signs and symptoms of malaria (22, 28). The remaining five studies assessed both ITN use and malaria knowledge (21–23, 29, 30). Only three studies used health behavior theories as frameworks for their respective interventions (29–31). Additionally, community involvement strategies were used in six studies ranging from exploring local knowledge and perceptions to developing action plans for health education interventions (22, 23, 29, 30), to entrusting trained residents to implement the intervention in their communities (25, 27).

### Study quality assessment

Based on the EPHPP Quality Assessment Tool, studies were rated on a scale of 1 (strong), 2 (moderate), to 3 (weak) for each category accordingly. These categories include selection bias (SB), study design (SD), confounders (C), blinding (B), data collection method (DCM), withdrawal and dropouts (W&DO), and overall quality of global rating (GR). A global rating of low, moderate, or high was assigned by averaging the six categories’ rankings. Studies without weak ratings across all categories were rated high quality in their final global rating. Studies of moderate quality have one category rated as weak, while those rated as low quality have a weak rating in two or more categories (Table 1). Five (*n* = 5) of the included studies were well-designed RCTs and c-RCTs, which provided detailed descriptions of the methods used and were assessed to be at low risk of bias (21–25). However, under the “blinding” subcategory, the risk of bias was deemed moderate for all studies, given that study participants were aware of their assignment to delivery strategies. Reports of study participants’ different characteristics at baseline were also noted in all studies, which could minimize potentially additional sources of bias. Five studies were classified as “high quality” (21–25) while five others were rated as “moderate (26–30).” Only one study (31) was considered low quality and therefore not included in the meta-analysis.

#### Impact of health education interventions

**Figure 2:**
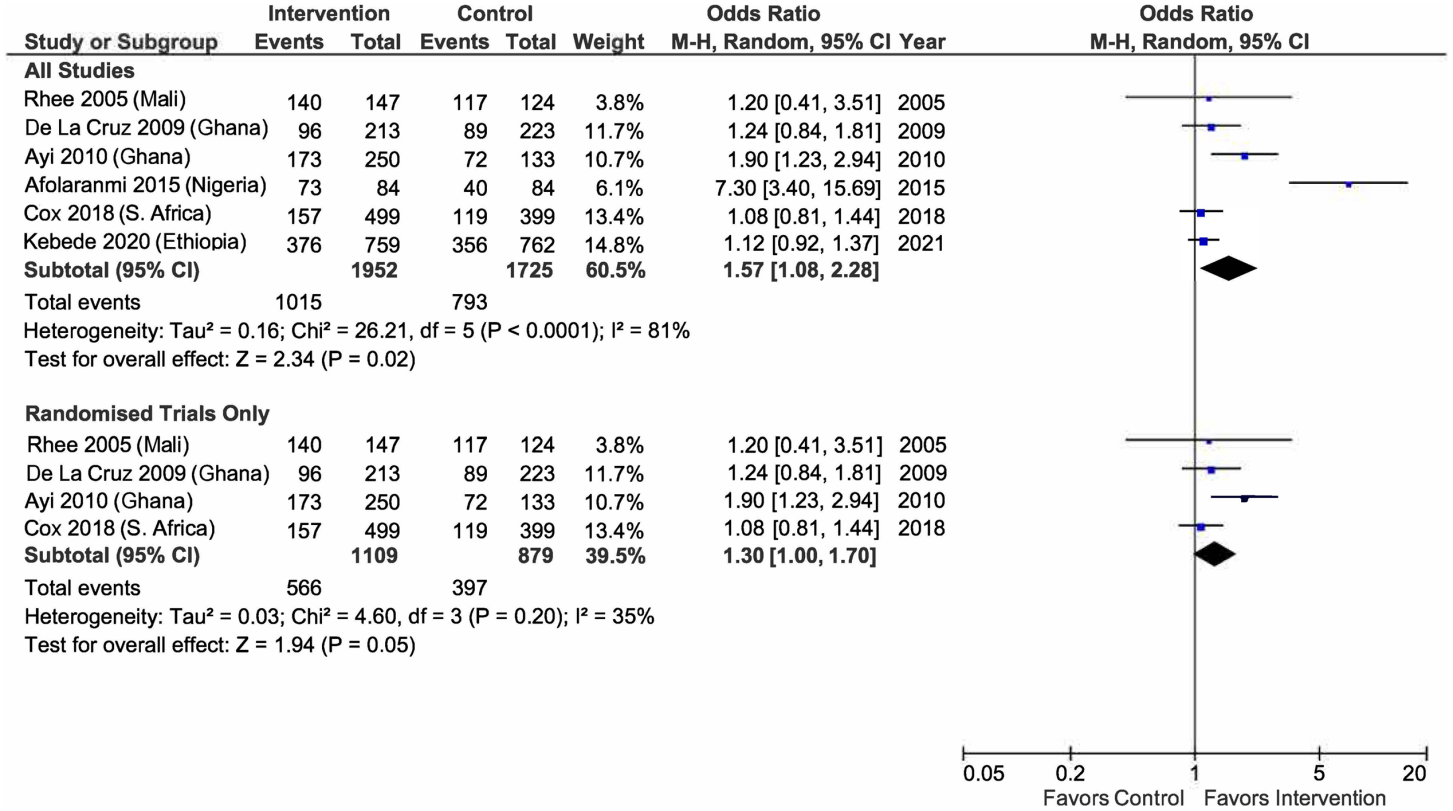
Odds of a person to answer malaria-related questions correctly between intervention and control group.

Two separate meta-analyses and synthesis of effect sizes were conducted for ten studies (*n* = 10) following their consistency in the types of interventions, study designs, and outcome variables (21–30). The first meta-analysis pooled estimates from six studies that assessed respondents’ malaria knowledge (21–24, 28, 30). These studies reported data from 3,704 participants (intervention *n* = 1,952, control *n* = 1,725). We found substantial heterogeneity between the studies with *T*^2^ value = 0.16 (*x*^2^ = 26.21, *df* = 5, *p* < 0.01) and *I*^2^ = 81%. A subgroup analysis of only randomized studies improved the heterogeneity to within the acceptable range (*I*^2^ = 35%), yielding a 95% prediction interval of the odds ratio of 1.00 to 1.70 (21–24). Our results found that, on average, health education modestly but significantly increases the odds of a person in the intervention group answering questions on malaria correctly compared to those in the control group (odds ratio 1.30, *p* = 0.05) (see Figure 2).

**Figure 3:**
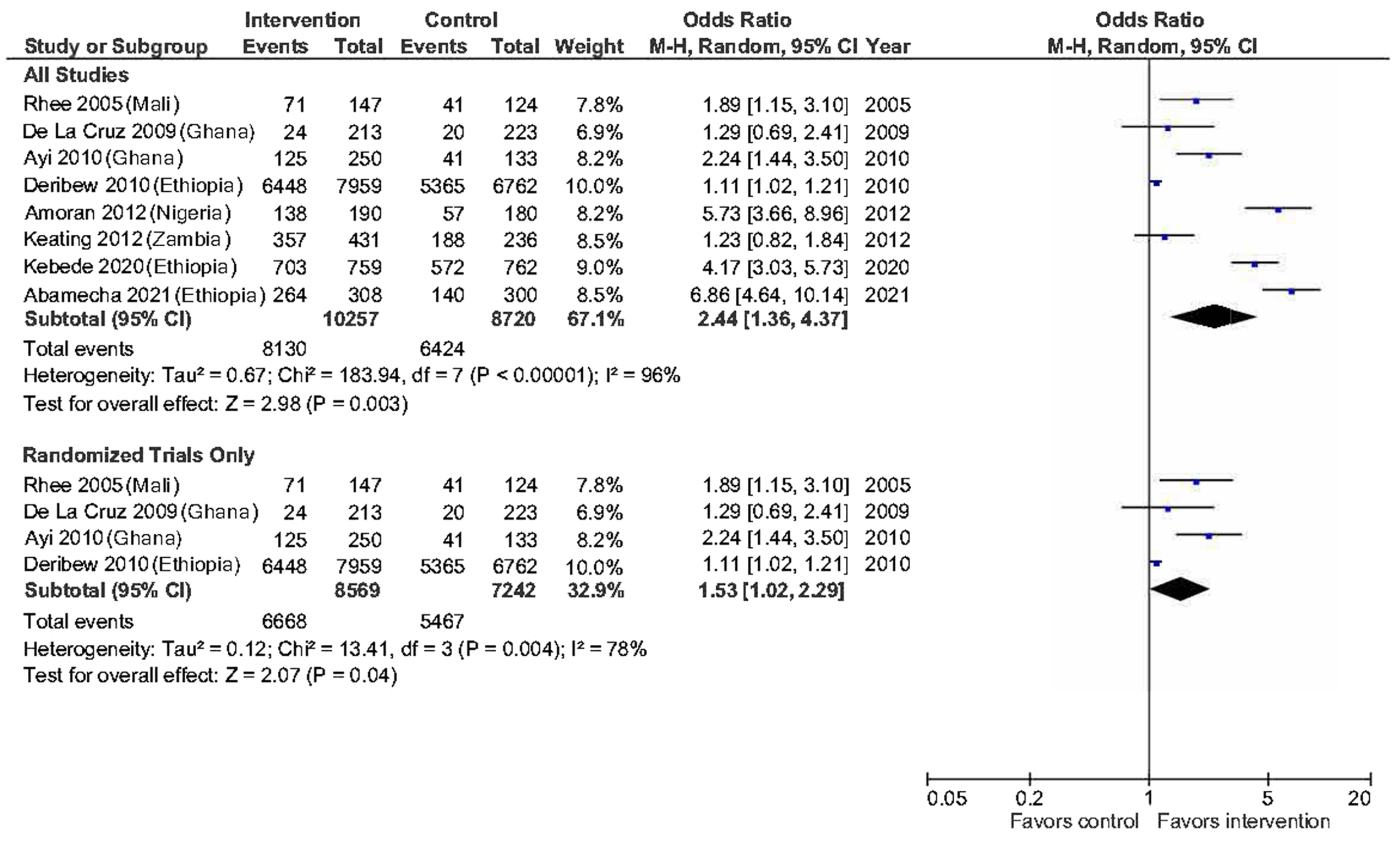
Odds of a person to use ITN between intervention and control group.

The second meta-analysis included eight studies that investigated ITN use among 18,977 respondents (intervention *n* = 10,257, control *n* = 8,720) (21, 23–27, 29, 30). Given the substantial heterogeneity between selected studies (*T*^2^ = 0.67, *x*^2^ = 183.94, *df* = 7, *p* < 0.01; *I*^2^ = 96%), a subgroup analysis of only randomized studies was carried out. We found that health education interventions significantly increase the odds of a person in the intervention group owning an ITN compared to those in the control group (odds ratio 1.53, 95% CI: 1.02 to 2.29, *p* = 0.004) with heterogeneity of *I*^2^ = 78% (see Figure 3). Additional exploratory subgroup analyses showed that the odds of ITN use substantially increased when the interventions were based on a model or framework (odds ratio 5.27, 95% CI: 3.24 to 8.58, *p* = 0.05) (27, 29) compared to when no model/framework was involved (odds ratio 1.86, 95% CI: 1.12 to 3.08, *p* < 0.01) (21, 23–26, 29).

## Discussion

This review identified published literature on health education interventions to improve malaria knowledge and ITN use. In general, the health education curriculum focused on the cause of malaria, ways of preventing malaria, types of mosquito nets, benefits of insecticide-treated bed nets, information on the importance of the consistent use of ITN, and steps to be taken to prevent malaria. A few of these interventions also provided demonstrations of proper ITN handling.

We evaluated 11 studies (i.e., 3 RCTs, 2 c-RCTs, 4 quasi-experimental, 1 pretest-postest, and 1 cross-sectional) (21–31) out of 683 found on seven databases. Selected studies were limited to those conducted among sub-Saharan African countries and populations. The duration of the interventions varied from 1 month to 5 years, with follow-up ranging from 2 weeks up to 5 years. From this review, it was found that each study used a curriculum that was specifically tailored to the study populations with varying delivery approaches.

On average, health education interventions significantly increased the odds of participants in the intervention groups reporting better malaria knowledge and a higher ITN usage rate than their counterparts. The findings remained statistically significant regardless of whether non-randomized studies were excluded or included. It is worth noting that the heterogeneity among the randomized studies was small, suggesting comparability in the intervention components and participants within the group. Therefore, the findings from the subgroup analysis can be generalized to situations and settings that resemble these studies (21–24). Our results also show that strategies adopted in the intervention arms could not address the knowledge-practice gap highlighted by many public health practitioners previously (39, 40). They argued that knowledge and attitudes towards a health threat would not necessarily translate into self-protective behavioral change, which was the case in our review of five studies that measured malaria knowledge and ITN use (21, 23, 24, 29, 30).

The impact of health education on ITN utilization was also comparable to other types of interventions to increase ITN usage conducted in the region. The most notable approach was the subsidization of ITN, in which participants would be offered a free ITN in the intervention groups. In contrast, those in the control groups would either pay a range of costs for an ITN (41–43), given microfinance loans (44, 45), or had to pay the full price (46). These studies reported odds ratios or risk ratios in the range of 0.66 (95% CI: 0.61 to 0.72) to 3.02 (95% CI: 2.78 to 3.30). A key exploratory finding was that the health education effect was significantly greater when a theory or model underpinned the interventions. This is consistent with the view that interventions based on behavioral theory are more efficacious in changing health-related behaviors than those that do not (47). This finding and all other findings from ITN utilization meta-analyses should be interpreted with caution owing to the high heterogeneity score and potential for confounders at the study level.

### Strength

To our knowledge, this is the first attempt at aggregating data on the impact of health education interventions on malaria knowledge and ITN use. The current systematic review and meta-analysis not only explore the strategies and approaches of each intervention but also provide a quantitative summary of the outcomes (i.e., malarial knowledge, ITN usage) from those studies. Additionally, these findings could aid policymakers, public health professionals, and other relevant stakeholders in deciding on the best practice to curb malaria in the sub-Saharan region.

### Study limitations

Our study has several limitations. The review only included English publications from recent years (i.e., from the year 2000), thus excluding useful information that may be found in publications of other languages and articles published before the year 2000. Our review excluded studies that were not conducted among the general population in sub-Saharan Africa, which may contain information that could be useful for the review. We were also unable to explore the influence of many potentially important factors such as the fidelity and sustainability of strategies used, duration and frequency of interventions, or the extent of relationships between the researchers, community health workers, and community members. Additionally, our results were only derived from published data which, most often than not, were “favorable” findings with positive results. Nonetheless, these findings present an opportunity for a broader debate about the impact of health education in health research.

## Conclusion

This review identified various strategies of health education interventions in sub-Saharan Africa. Methods utilized to educate the community on malaria have improved their knowledge and ITN uptake. While it was not possible to draw conclusions on which strategies, duration, or frequency of educational interventions were the best, these interventions are valuable given that ITNs are still the region’s primary vector control method (48). Factors such as merging training with interpersonal communication, the community taking active roles in intervention efforts, and using evidence-based health educational messages have contributed to the global effort in combating malaria in sub-Saharan countries. Overall, it is concluded that intervention strategies of health education were moderately effective in improving malaria knowledge and ITN usage.

## Data availability statement

The original contributions presented in the study are included in the article/Supplementary material, further inquiries can be directed to the corresponding author.

## Author contributions

OO and AMN were the major contributors in preparing the manuscript and analyzed and interpreted the data. SI is OO’s PhD supervisor at the time of manuscript preparation and has contributed significantly to shaping the direction of this paper. All authors contributed to the article and approved the submitted version.

## Conflict of interest

The authors declare that the research was conducted in the absence of any commercial or financial relationships that could be construed as a potential conflict of interest.

## Publisher’s note

All claims expressed in this article are solely those of the authors and do not necessarily represent those of their affiliated organizations, or those of the publisher, the editors and the reviewers. Any product that may be evaluated in this article, or claim that may be made by its manufacturer, is not guaranteed or endorsed by the publisher.
