# Supplementary material for: Effectiveness of health education interventions to improve malaria knowledge and insecticide-treated nets usage among populations of sub-Saharan Africa: systematic review and meta-analysis
Source: Front Public Health. 2023 Aug 3;11:1217052. doi: 10.3389/fpubh.2023.1217052 (PMC10435857; doi:10.3389/fpubh.2023.1217052)
Supplement: Supplementary file 1 [file Data_Sheet_1.PDF]

**Inclusion criteria**	**Search Terms**
1. Population of sub-Saharan African countries	Africa*<br>“Sub-Saharan*”<br>names of sub-Saharan African countries (40 countries)
2. The studies must have carried out malaria intervention or report pre- and post-test results.	“malaria intervention*”<br>evaluat*<br>impact*<br>effectiveness<br>“malaria prevention*”<br>health education*
3. There must be a comparison group, or two-related groups	“control group*”<br>“comparison group*”<br>“intervention group*”<br>“matched group*”
4. Research design- we included both RCT and non-RCT studies e.g., quasi-experiments, cross-sectional.	RCT<br>random*<br>treatment<br>experiment*<br>quasi experiment*<br>cross section*
5. Primary outcome measures – malaria knowledge or ITN use or both.	“malaria knowledge*”<br>knowledge*<br>“ITN use*”<br>“LLTN use*”<br>“LLIN use*”<br>“mosquito net use*”<br>“bed net use*”
6. Timeframe – 2000 to 2021	2000 to 2021
7. Publication status – Published	
8. Language	English
