# Supplementary material for: Effectiveness of health education interventions to improve malaria knowledge and insecticide-treated nets usage among populations of sub-Saharan Africa: systematic review and meta-analysis
Source: Front Public Health. 2023 Aug 3;11:1217052. doi: 10.3389/fpubh.2023.1217052 (PMC10435857; doi:10.3389/fpubh.2023.1217052)
Supplement: Supplementary file 2 [file Data_Sheet_2.PDF]

Abstract Screening Guide

Citation, Title, and Abstract Screening

1. Does the **citation** indicate publication on or after 2000?
    a. Yes: continue screening
    b. No: stop screening

2. Does the **title or abstract** written in English?
    a. Yes: continue screening
    b. No: stop screening

3. Does the **title or abstract** indicate that a malaria health education was conducted?
    a. Yes: continue screening
    b. No: stop screening

4. Does the **title or abstract** indicate that this is NOT a correction or erratum?
    a. Yes: continue screening
    b. No: stop screening

Abstract Screening

5. Does the **abstract** indicate that the study report pre- and post-test results?
    a. Yes or Unsure/Unclear: continue screening
    b. No: stop screening

6. Does the **abstract** indicate that malaria knowledge or ITN use was the outcome being studied?
    a. Yes or Unsure/Unclear: continue screening
    b. No: stop screening

7. Does the **abstract** indicate that the study uses a quantitative design?
    a. Yes or Unsure/Unclear: continue screening

        -Key words: regression, covariate, modeling, structural equation modeling, mean, standard deviation, correlation, variance, causal, experiment, QED, randomized controlled trial (RCT), propensity score matching, Quasi-experimental

    b. No: stop screening

        -For example: qualitative only: ethnography, action research, social observation, focus groups, case study research

Decision: **Should this article be included?**

a. **Yes**, all 7 screening questions answered Yes or Unclear

b. **No**, at least one answers definitely "No"
